# Detecting vibration source for the orientation behavior of sand scorpions

**DOI:** 10.1186/1471-2202-13-S1-P106

**Published:** 2012-07-16

**Authors:** Eunseok Jeong, DaeEun Kim

**Affiliations:** 1 Biological Cybernetics, School of Electrical and Electronic Engineering, Yonsei University, Shinchon, Seoul, 120-749, South Korea

## 

Sand scorpions can locate their prey through their vibration sensitivity. They have their tactile sense organs on their legs to detect vibration reaching their body. They show an orientation behavior towards their prey when there is any vibration signal generated by prey movement. How they respond to the vibration source is an open question. It is believed that stimulus-locked neuron firings from the sensor organs on each leg are processed and the brain system of sand scorpions has sensory projections from the sense organs. It is known that eight command neurons in the brain interact each other with triad inhibitions [1,2]. Then a population coding of the neuron activity determines the direction of vibration source [3].

**Figure 1:**
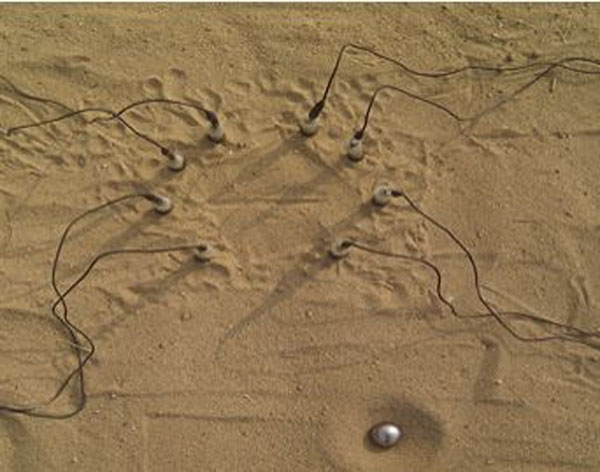
Experimental setup in the sand

We tested direction selectivity in the sand with an experimental setup to detect the vibration source as shown in Figure 1. Eight legs of sand scorpions are positioned almost in a rim of circle. We placed eight microphones in a circle and each microphone sensor detects the vibration signal. The positions of microphone sensors are similar to the foot positions of sand scorpions, that is, the same angular positions (at 18, 54, 90, 140, -140, -90, -54, and -18 degrees). A metal ball is dropped to produce disturbance vibration. Then the time course of vibration signals is received by each microphone sensor. The time delay of vibration for each leg is a prominent source to detect the direction of vibration. We observe P-wave and Rayleigh wave of vibration signals, where the P-wave corresponds to a compressional sound wave and Rayleigh wave is a surface wave easily found in the sand. Both P-wave and Rayleigh wave can provide a cue to detect direction of vibration source. Yet the Rayleigh wave can be observed with large amplitude while the P-wave with small amplitude is sensitive to noise.

In our experiments, sensory activations depending on Rayleigh wave are measured with microphone sensors and a population coding of command neuron activities with triad inhibitions are applied to the time course of vibration sensor readings. The approach can estimate the direction of vibration source. Unlike the direction, the distance to a vibration source is not clearly measured in our experiments. The distance estimation seems to be involved with the intensity of vibration signals or the time difference between the P-wave and the Rayleigh wave. The time difference roughly guides the distance in the experiments, but it does not provide accurate results. It suggests that sand scorpions might focus on estimating the direction of the vibration source caused by a prey rather than accurately calculate how far away from their body a prey is. The distance accuracy may be achieved within small distances.

Sand scorpions can orient towards their prey with high accuracy when there is any prey movement. We assume a circular array of directionally sensitive neurons with inhibition mechanism to explain the orientation behavior, receiving sensory signals of vibration as pointed out by other researchers [1,2]. What type of inhibition mechanism is available, how they detect the distance of a vibration source, or whether they can sense or use both the sound wave and surface wave is an open question. We need further study on these subjects. More sophisticated measurements and experiments of vibration signals might explain those questions partly.
